# Low Level of Serum Immunoglobulin G Is Beneficial to Clinical Cure Obtained With Pegylated Interferon Therapy in Inactive Surface Antigen Carriers

**DOI:** 10.3389/fimmu.2022.864354

**Published:** 2022-04-22

**Authors:** Hong Li, Xiao Lin, Lili Liu, Ling Qin, Yanhong Zheng, Xiaohui Liu, Xinhuan Wei, Shan Liang, Yali Liu, Jing Zhang, Xinyue Chen, Zhenhuan Cao

**Affiliations:** ^1^ The Third Unit, Department of Hepatology, Beijing Youan Hospital, Capital Medical University, Beijing, China; ^2^ The First Unit, Department of Hepatology, Beijing Youan Hospital, Capital Medical University, Beijing, China; ^3^ Biomedical Information Center, Beijing Youan Hospital, Capital Medical University, Beijing, China

**Keywords:** inactive HBsAg carriers, HBsAg clearance, pegylated interferon, lgG, predictor

## Abstract

**Purpose:**

Our recent study showed a high rate of HBsAg clearance in inactive HBsAg carriers (IHCs) treated with pegylated IFN (PEG-IFN). To better understand the immune-mediated component of HBsAg clearance, this study investigated the role of serum immunoglobulin G (IgG) and its subclasses in predicting HBsAg clearance in IHCs with PEG-IFN therapy.

**Methods:**

In this study, IHCs received PEG-IFN for 96 weeks. Subjects who achieved clearance of HBsAg were considered responders (R group), and those in whom HBsAg was not cleared were considered non-responders (NR group). The HBsAg, ALT, and serum lgG subtypes (lgG1, IgG2, IgG3, lgG4) were tested at baseline, and at 12 and 24 weeks of treatment. To evaluate the factors in predicting HBsAg clearance, univariate and multivariate logistic regression analyses were performed. The receiver operator characteristic curves and the area under the receiver operator characteristic curve (AUROC) were used to evaluate prognostic values.

**Results:**

Our results showed that 39 cases obtained HBsAg clearance (group R), while 21 cases did not (group NR). There was no significant difference in age, ALT, and AST levels between the two groups. The serum levels of IgG1, lgG2, lgG3 and lgG4 at baseline, and at 12 and 24 weeks were significantly lower in IHC with HBsAg clearance than in the NR group. Univariate logistic regression analysis showed that serum IgG1, IgG2, IgG3, and IgG4 levels at baseline, and at 12, and 24 weeks were all strong predictors of HBsAg clearance. In all indicators, lgG2 had the highest AUROC at baseline and lgG3 the highest AUROC at week 12. A multifactor logistic analysis was performed with y=33.933-0.001*BaselinelgG1-0.002*BaselinelgG2. The area under the curve was 0.941 with 100% sensitivity and 76.19% specificity.

**Conclusion:**

Together, our findings suggest that serum IgG has a higher predictive value compared to the convention predictors of HBsAg and ALT for HBsAg clearance and thus may be a better clinical predictor of HBsAg clearance in IHCs.

## Introduction

Chronic hepatitis B virus (HBV) infection is a major public health problem in China with approximately 70 million cases (1). Hepatitis B surface antigen (HBsAg) clearance, although a desirable therapeutic goal, is extremely difficult to obtain clinically. Research into the mechanism of HBsAg clearance is severely hampered by the lack of specimens from HBsAg-cleared patients. According to recent reports, Inactive surface antigen carriers (IHCs) are a large population that accounts for approximately 36% of patients with HBV infection (2) and amount to 30 million individuals in total (3). IHCs are defined by normal alanine aminotransferase (ALT), HBV DNA ≤2000 IU/mL, and hepatitis B e antigen (HBeAg)-negative status. Our and other recent studies have shown that IHC treated with pegylated-interferon (PEG-IFN) results in high HBsAg clearance, with HBsAg clearance rates of 44.7% to 65% (4–6). In addition to cellular immunity, humoral immunity also plays an important role in HBV infection and elimination. Immunoglobulin G (lgG) is the main component of serum immunoglobulins and is classified into four isoforms, lgG1, lgG2, lgG3, and lgG4, depending on the amino acid composition and structure of the hinge region (7). Serum lgG and subclasses play a central role in the humoral immune response. IgG1 is the most abundant lgG subclass in the human serum, followed by lgG2, lgG3, and lgG4. IgG1 mediates the immune response to pathogens, binding to soluble and membrane protein antigens through its variable region, while activating the effector mechanisms of the innate immune system, binding to C1q to cause complement-dependent cytotoxicity and binding to each of the different Fc receptors to cause antibody-dependent cell-mediated cytotoxicity (8). lgG3 appears early in the infection and may limit the excessive inflammatory response (9). lgG4 usually appears in a non-infected setting or after prolonged and repeated exposure to antigens and is associated with lgG4-related diseases, involving multiple organs and tissues in chronic progressive autoimmune disorders (10), which were patients excluded from this study. Recent studies have reported that lgG may be involved in the onset and development of HBV infection, that the severity of HBV pathogenesis is related to immune function of the body, and that serum IgG levels are significantly higher in patients with chronic liver failure and severe chronic hepatitis B than in patients with mild to moderate disease and are positively correlated with the severity of the disease (11, 12).

However, the role of IgG in antiviral therapy, particularly in HBsAg clearance, has not been reported. IHCs were recruited in this study and were treated with PEG-IFN for 96 weeks. We detected the levels of serum lgG and its subtypes during treatment and investigated their value in predicting the clearance of HBsAg.

## Materials and Methods

### Patients

A total of 60 IHCs were recruited for the study and received regular follow-up visits at Beijing Youan Hospital, Capital Medical University. Five healthy students were recruited as healthy controls.

All IHCs met the criteria defined in the prevention and treatment guidelines for chronic hepatitis B (2019 edition) (1) (i) HBsAg positive > 6 months and HBsAg <1000 IU/mL, HBeAg negative, anti-HBe positive/positive; (ii) HBV DNA <2000 IU/mL, ALT normal (male<50 IU/L, female<40 IU/L); (iii) white blood cell count >4×10^9^/L, platelet count >150×10^9^/L; (iv) total bilirubin <34 μmol/L, albumin >40 g/L. Exclusion criteria: (i) history of autoimmune diseases; (ii) Human immuno-deficiency virus or Hepatitis C Virus or Hepatitis E Virus coinfection; (iii) Pregnant, lactating women and those who are preparing to have children soon; (iv) liver cirrhosis or liver cancer; (v) history of severe heart disease, including unstable or uncontrolled heart disease within 6 months; (vi) history of mental illness or psychiatric disorders. (vii) Uncontrolled epilepsy. (viii) Unabated alcohol or drug abuser. (ix) Uncontrolled diabetes mellitus, hypertension, thyroid disease, retinopathy; (x) contraindication to interferon. All healthy controls were HBsAg-negative and anti-HBs -positive.

### Treatment and Efficacy

The 60 patients enrolled in the study were treated with PEG-IFN 135 μg weekly by subcutaneous injection. Treatment was stopped if neutrophil counts were <0.50×10^9^ or platelet count was <25×10^9^, or a serious adverse events occurred. The total duration of treatment was 96 weeks. The effect of the treatment was determined by HBsAg clearance. Subjects who achieved HBsAg clearance in 96 weeks were considered responders (group R), and those in whom HBsAg was not cleared were considered non-responders (group NR).

### Ethics Approval

The protocol and the consent form for the study were approved by the research ethics committee of the Beijing You’an Hospital, Capital Medical University, China ([2017]24).

### Laboratory Tests

Blood samples were collected at baseline, and after 12, and 24 weeks of treatment and were tested for HBV DNA, HBsAg levels, liver function, and routine blood tests. Serum levels of the lgG subtypes (lgG1, IgG2, IgG3, lgG4) were also measured simultaneously. HBV DNA was quantified using the fluorescence quantitative (FQ)-PCR, Cobas Taqman real-time polymerase chain reaction 2.0 system (Roche, Germany), with a lower limit of detection of 20 IU/mL. HBsAg quantification was performed using the HBsAg quantification kit from Roche, having a lower limit of detection of 0.05 IU/mL. Liver function testing was performed by reagents from Shanghai Kehua Dongling Company (China). lgG subtypes were detected by enzyme-linked immunosorbent assay kits from Jianglai Biologicals (China). In order to measure the concentration of IgG subtypes in the sample, this IgG ELISA Kit includes a set of calibration standards. The calibration standards are assayed at the same time as the samples and allow the operator to produce a standard curve of Optical Density versus IgG subtypes concentration. The concentration of IgG subtypes in the samples is then determined by comparing the O.D. of the samples to the standard curve.

### Statistical Analysis

Data analysis was performed using SPSS 25 software (IBM SPSS, Chicago, IL, USA), and values were expressed as mean ± standard deviation (SD) and median (25th, 75th), The Mann–Whitney U test or Student’s t-test was applied for quantitative variables, and the chi-square or Fisher’s exact test was used for categorical variables. Receiver operator characteristic (ROC) curves, which plot sensitivity by 1 – specificity, and the area under the ROC curve (AUROC) were used to evaluate the prognostic values of the quantitative HBsAg, ALT, IgG1, IgG2, IgG3, IgG4 at baseline, and at weeks 12 and 24 as well as the HBsAg change form baseline at weeks 12 and 24 to predict HBsAg clearance. Univariate and multivariate logistic regression analyzes were performed to evaluate the magnitude and significance of the association. A two-sided P-value <0.05 was considered statistically significant.

## Results

### Levels of Serum IgG1, IgG2, IgG3, and IgG4 in IHCs and Healthy Subjects

**Figure 1:**
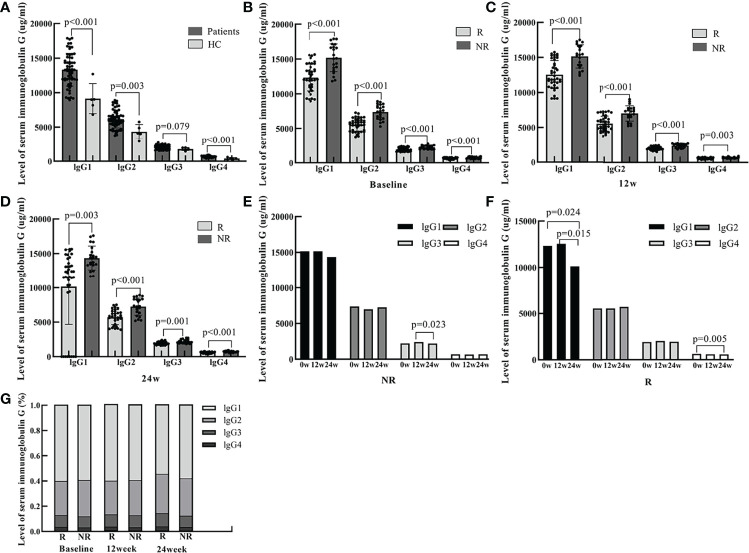
The serum levels of IgG1, IgG2, IgG3, and IgG4 at baseline and 12 and 24 weeks of PEG-IFN treatment in IHCs and healthy controls. **(A)** The level of lgG1, lgG2, lgG3, lgG4 were significantly higher in IHCs than in healthy controls (p<0.001, p=0.003, p=0.079, p<0.001, respectively). **(B)** The levels of serum lgG1, lgG2, lgG3, and lgG4 were significantly lower in the R group than in the NR group at baseline (p<0.001, p<0.001, p<0.001, p<0.001, respectively). **(C)** The levels of serum lgG1, lgG2, lgG3, and lgG4 were significantly lower in the R group than in the NR group at week 12 of PEG-IFN treatment (p<0.001, p<0.001, p<0.001, and p=0.003, respectively). **(D)** The serum levels of lgG1, lgG2, lgG3, and lgG4 were significantly lower in the R group than in the NR group at week 24 of PEG-IFN treatment (p=0.003, p<0.001, p=0.001, and p<0.001, respectively). **(E)** The change in the serum levels of lgG1, lgG2 and lgG4 were not significant in the NR group. The serum levels of IgG3 was lower at week 24 of PEG-IFN treatment than at 12 weeks (p=0.023). **(F)** The serum levels of IgG1 were lower at week 24 of PEG-IFN treatment than at baseline and at 12 weeks (p=0.024 and p=0.015, respectively). **(G)** The proportion of lgG1, lgG2, lgG3 and lgG4 were not significant in the R and NR group.

Sixty IHCs (43 males and 17 females with a mean age of 38.95 ± 11.23 years) and five healthy controls with a mean age of 27.60 ± 1.67 years were enrolled. lgG1, lgG2, lgG3, and lgG4 levels were 13328.17 μg/mL, 6186.79 μg/mL, 2024.53 μg/mL, and 660.76 μg/mL in IHCs and 9144.00 μg/mL, 4342.61 μg/mL, 1772.73 μg/mL, 395.17 μg/mL, respectively in healthy controls. The levels of lgG1, lgG2, lgG3, and lgG4 were significantly higher in IHC patients than in healthy controls. (p<0.001, p=0.003, p=0.079, p<0.001, respectively) (**Figure 1**).

### The Level of IgG Subtype at Baseline and During Treatment in R and NR Group

A total of 60 patients enrolled in the study were treated with PEG-IFN for 96 weeks. Of these, 39 cases achieved HBsAg clearance (group R) and 21 cases did not (group NR). There was no statistical difference in age, or in ALT and AST values between the two groups. HBsAg quantification at baseline, at 12 and 24 weeks were all lower in the R group than in the NR group (*P*=0.067, *P*=0.001, *P*<0.001). In addition, the levels of serum immunoglobulin IgG1, lgG2, lgG3, and lgG4 at baseline, 12 and 24 weeks were all significantly lower in patients with HBsAg clearance than in the NR group (**Table 1** and **Figure 1**)

**Table 1:** Characteristics of R and NR groups at baseline, and after 12 and 24 weeks of treatment.

Parameter	R group	NR group	*P*-value
	n=39	n=21	
Sex (M/F)	27/12	16/5	0.568
Age (years)			0.368
Mean ± SD	38.05 ± 11.25	40.62 ± 11.26	
Median (25th, 75th)	37 (30, 46)	38 (31, 53)	
Baseline			
ALT (U/L)			0.570
Mean ± SD	30.88 ± 14.62	33.17 ± 14.36	
Median (25th, 75th)	27.00 (17.7, 47.00)	37.00 (17.75, 47.25)	
AST (U/L)			0.946
Mean ± SD	27.97 ± 6.89	28.17 ± 8.32	
Median (25th, 75th)	26.50 (24.00, 35.00)	28.00 (20.75, 36.25)	
Ig HBsAg (IU/mL)			0.067
Mean ± SD	1.61 ± 0.97	2.08 ± 0.73	
Median (25th, 75th)	1.70 (1.00, 2.38)	2.27 (1.60, 2.72)	
lgG1 (μg/mL)			<0.001
Mean ± SD	12338.97 ± 1930.58	15165.23 ± 1995.97	
Median (25th, 75th)	12180.00 (10970.00, 13830.00)	15100 (13565, 16915)	
lgG2 (μg/mL)			<0.001
Mean ± SD	5550.00 ± 924.60	7369.41 ± 1081.19	
Median (25th, 75th)	5640.00 (4593.96,6219.33)	7503.63 (6452.96, 8349.91)	
lgG3 (μg/mL)			<0.001
Mean ± SD	1915.80 ± 282.70	2226.45 ± 248.62	
Median (25th, 75th)	1866.95 (1655.60, 2225.73)	2190.93 (1972.52, 2430.58)	
lgG4 (μg/mL)			<0.001
Mean ± SD	635.69 ± 105.68	707.32 ± 138.61	
Median (25th, 75th)	653.56 (552.65, 717.16)	666.74 (573.92, 846.55)	
12 week			
ALT (U/L)			0.681
Mean ± SD	82.56 ± 89.52	66.05 ± 49.14	
Median (25th, 75th)	47.00 (34.00, 96.00)	46.00 (34.50, 81.00)	
AST (U/L)			0.545
Mean ± SD	64.89 ± 74.50	47.71 ± 28.05	
Median (25th, 75th)	45.00 (32.00, 60.00)	42.00 (27.50, 56.00)	
Ig HBsAg (IU/mL)			0.001
Mean ± SD	0.67 ± 1.21	1.70 ± 0.98	
Median (25th, 75th)	1.10 (-0.47, 1.68)	1.70 (1.34, 2.40)	
lgG1 (μg/mL)			<0.001
Mean ± SD	12,559.74 ± 2002.66	15,156.19 ± 1657.72	
Median (25th, 75th)	11,980.00 (11,030.00, 14,740.00)	15,390.00 (13,725.00, 16,665.00)	
lgG2 (μg/mL)			<0.001
Mean ± SD	5537.06 ± 1083.08	6989.41 ± 1103.60	
Median (25th, 75th)	5380.72 (4573.19, 6395.55)	7284.68 (6084.49, 7731.92)	
lgG3 (μg/mL)			<0.001
Mean ± SD	2027.03 ± 248.77	2417.47 ± 282.86	
Median (25th, 75th)	2001.42 (1824.82, 2225.53)	2489.63 (2141.71, 2651.88)	
lgG4 (μg/mL)			0.003
Mean ± SD	589.99 ± 107.16	679.47 ± 100.57	
Median (25th, 75th)	589.97 (492.85, 675.43)	654.71 (586.96, 743.95)	
24 week			
ALT (U/L)			0.728
Mean ± SD	54.77 ± 50.70	54.45 ± 48.95	
Median (25th, 75th)	37.00 (26.00, 69.00)	37.50 (28.50, 61.50)	
AST (U/L)			0.636
Mean ± SD	46.39 ± 28.24	46.65 ± 45.28	
Median (25th, 75th)	36.00 (28.00, 55.00)	33.50 (31.00, 38.75)	
Ig HBsAg (IU/mL)			<0.001
Mean ± SD	-0.29 ± 1.14	1.22 ± 1.27	
Median (25th, 75th)	-0.03 (-0.92, 0.45)	1.50 (0.62,2.35)	
lgG1 (μg/mL)			0.003
Mean ± SD	10146.92 ± 5464.79	14351.90 ± 1744.12	
Median (25th, 75th)	12,190.00 (9430.00, 13,580.00)	14,420.00 (13,325.00, 15,525.00)	
lgG2 (μg/mL)			<0.001
Mean ± SD	5714.71 ± 1079.45	7252.18 ± 12.61.89	
Median (25th, 75th)	5810.81 (4684.04, 6566.44)	7289.20 (6111.19, 8326.01)	
lgG3 (μg/mL)			0.001
Mean ± SD	1943.30 ± 222.42	2202.62 ± 287.04	
Median (25th, 75th)	1903.45 (1771.35, 2109.15)	2089.65 (1940.83, 2485.29)	
lgG4 (μg/mL)			<0.001
Mean ± SD	579.33 ± 110.13	716.37 ± 101.61	
Median (25th, 75th)	567.05 (522.30, 665.69)	713.72 (648.41, 793.03)	

### HBsAg Changes in IHC Patients After PEG-IFN Treatment

**Figure 2:**
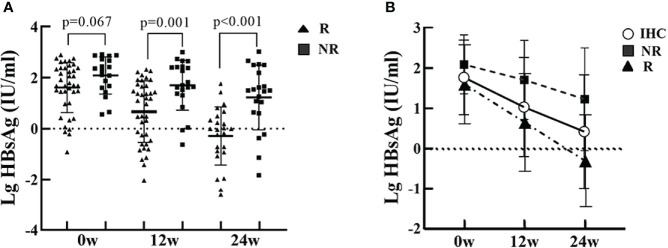
HBsAg at baseline, and at 12 and 24 weeks of PEG-IFN treatment in the R and NR groups. **(A)** The level of HBsAg in the R group was lower than in the NR group, but were not significantly different (P=0.067). The levels of HBsAg in the R group was significantly lower than in the NR group (P=0.001, P<0.001) at 12 and 24 weeks of treatment. **(B)** HBsAg showed a significant downward trend after PEG-IFN treatment in IHCs, with HBsAg quantification at 12 and 24 weeks significantly lower than baseline, especially in the R group.

The baseline HBsAg quantification in group R was not significantly different from group NR. After PEG-IFN treatment, HBsAg showed a significant downward trend, with HBsAg quantification at 12 and 24 weeks significantly lower than baseline, especially in patients in group R. HBsAg quantification at 12 and 24 weeks was significantly lower in group R than in group NR (*P*=0.001, *P*<0.001) (**Figure 2**).

### The Value of Serum IgG on HBsAg Clearance

To evaluate factors at identifiable at baseline or in early treatment able to predict HBsAg clearance, univariate logistic regression analysis was conducted. The variables included in the analysis were HBsAg, ALT, IgG1, IgG2, IgG3, and IgG4 at baseline, 12, and 24 weeks as well as HBsAg change form baseline at weeks 12 and 24. The results showed that the serum levels of IgG1, IgG2, IgG3, and IgG4 at baseline, 12, and 24 weeks were all strong predictors of HBsAg clearance as well as HBsAg levels at 12 and 24 weeks, HBsAg changes from baseline and at 12 and 24 weeks. Sex, age, baseline HBsAg and ALT levels were not statistically significant (**Table 2**).

**Table 2:** Variables at baseline and early treatment associated with HBsAg clearance (univariable analysis).

Variable	OR	95% CI	*P*-value
Sex	0.32	(0.423, 4.783)	0.569
Age	0.72	(0.934, 1.027)	0.397
Baseline HBsAg	3.17	(0.247, 1.070)	0.075
Baseline ALT	0.82	(0.987, 1.005)	0.366
Baseline lgG1	13.80	(0.999, 1.000)	<0.001
Baseline lgG2	13.98	(0.997, 0999)	<0.001
Baseline lgG3	11.37	(0.994, 0.998)	0.001
Baseline lgG4	4.44	(0.990, 1.000)	0.035
Week 12 HBsAg	8.15	(0.215, 0.752)	0.004
Week 12 HBsAg change from baseline	5.47	(1.237, 11.234)	0.019
Week 12 ALT	0.59	(0.995, 1.012)	0.442
Week 12 IgG1	13.61	(0.999, 1.000)	<0.001
Week 12 lgG2	13.22	(0.998, 0.999)	<0.001
Week 12 lgG3	15.16	(0.992, 0.997)	<0.001
Week 12 lgG4	7.46	(0.986, 0.998)	0.006
Week 24 HBsAg	9.74	(0.175, 0.671)	0.002
Week 24 HBsAg change from baseline	10.72	(1.772, 9.764)	0.001
Week 24 ALT	0.001	(0.989, 1.012)	0.982
Week 24 IgG1	7.68	(0.999, 1.000)	0.006
Week 24 lgG2	11.67	(0.998, 1.000)	0.001
Week 24 lgG3	8.81	(0.993, 0.999)	0.003
Week24 lgG4	10.84	(0.980, 0.995)	0.001

### Value of Baseline Variables for Predicting HBsAg Clearance

**Figure 3:**
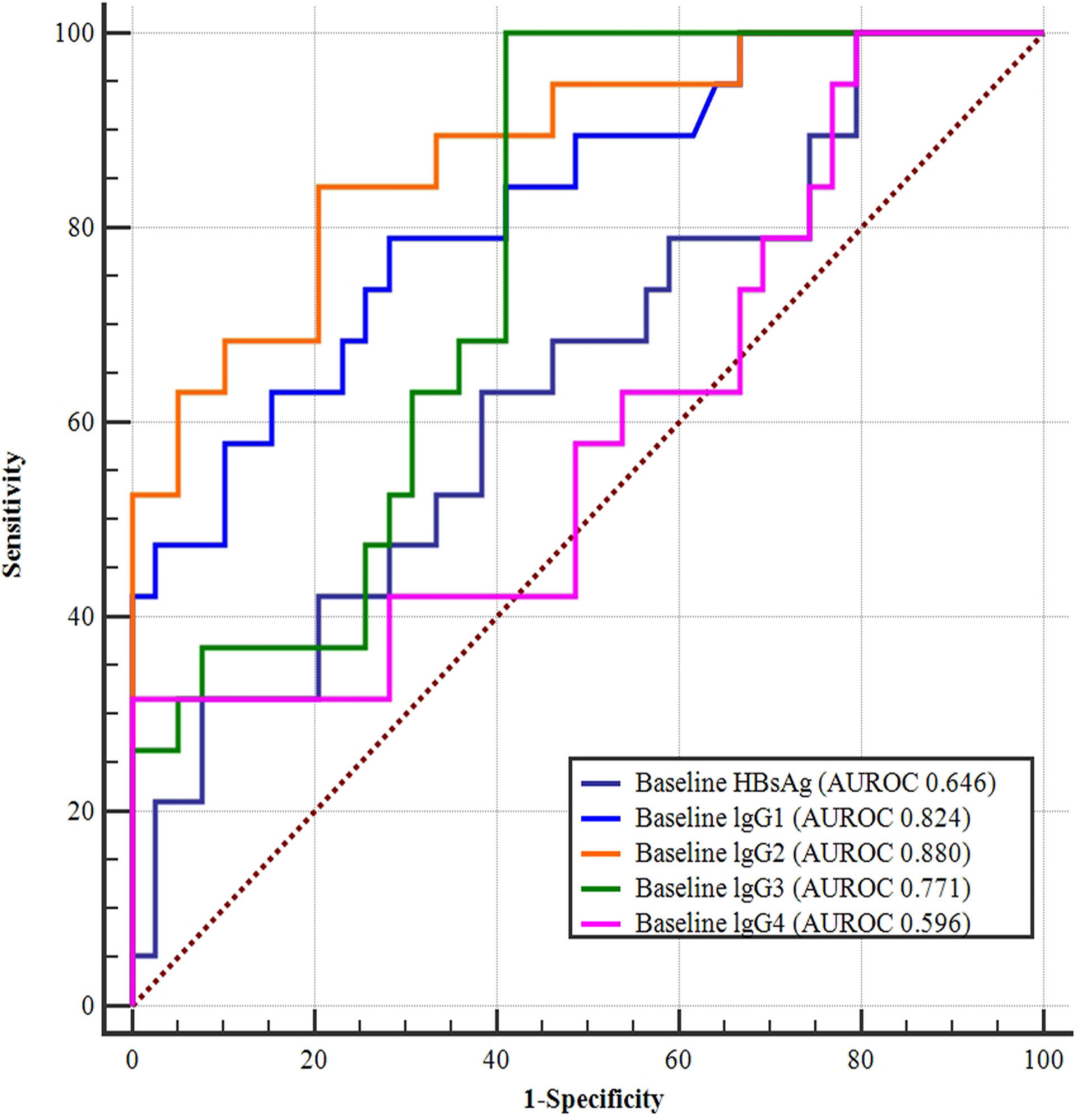
ROC curves of baseline HBsAg, IgG1, IgG2, IgG3, and IgG4 levels for predicting HBsAg clearance. Baseline lgG2 had the largest area under the curve (AUROC 0.880), followed by the baseline lgG1 (AUROC 0.824), lgG3 (AUROC 0.771), HBsAg (AUROC 0.646), and lgG4 (AUROC 0.596) levels.

**Figure 4:**
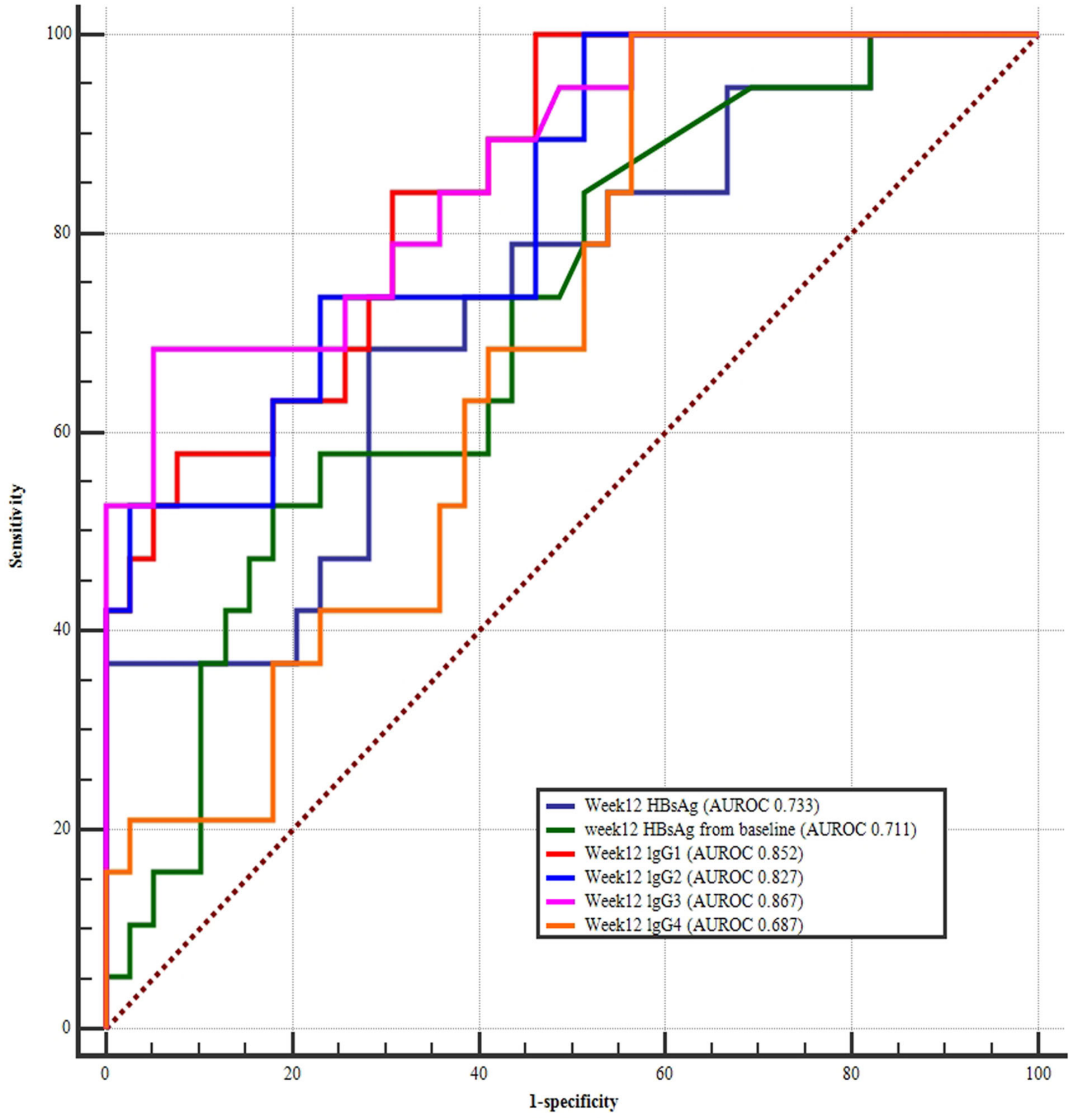
ROC curves at week 12 HBsAg, and week 12 HBsAg changes from baseline, week 12 IgG1, IgG2, IgG3, and IgG4 for predicting HBsAg clearance. The largest area under the curve was week 12 lgG3 (AUROC 0.867), followed by week 12 IgG1 (AUROC 0.852), week 12 lgG2 (AUROC 0.827), week 12 HBsAg (AUROC 0.733), and HBsAg change form baseline (AUROC 0.711).

In the univariate analysis, the *P*-values for baseline HBsAg, lgG1, lgG2, lgG3, and lgG4 were all less than 0.1; the respective ROC curves for predicting HBsAg clearance are shown in **Figure 4**. The results suggest that baseline lgG2 values had the largest area under the curve (AUROC 0.880), followed by the baseline levels of lgG1 (AUROC 0.824), lgG3 (AUROC 0.771), HBsAg (AUROC 0.646), and lgG4 (AUROC 0.596). The areas under the ROC curves were further compared and the predictive value of baseline IgG2 was significantly higher than baseline HBsAg (**Figure 3** and **Table 3**).

**Table 3:** Comparison of the areas under the ROC curves for each indicator at baseline and 12 weeks, 24 weeks.

Indicator of baseline	Difference between areas	95% CI	*P*
Baseline lgG1 *vs*. Baseline HBsAg	0.18	-0.026, -0.381	0.09
Baseline lgG2 *vs*. Baseline HBsAg	0.23	0.053, -0.414	0.01
Baseline lgG3 *vs*. Baseline HBsAg	0.12	-0.067, -0.315	0.20
Baseline lgG4 *vs*. Baseline HBsAg	0.05	-0.179, -0.279	0.67
Week 12 lgG1 *vs*. Week 12 HBsAg change from baseline	0.14	-0.029, -0.311	0.10
Week 12 lgG2 *vs*. Week 12 HBsAg change from baseline	0.18	-0.082, -0.315	0.25
Week 12 lgG3 *vs*. Week 12 HBsAg change from baseline	0.16	0.003, -0.310	0.04
Week 12 lgG4 *vs*. Week 12 HBsAg change from baseline	0.02	-0.164, -0.211	0.80
Week 12 lgG1 *vs*. Week 12 HBsAg	0.12	-0.068, -0.305	0.21
Week 12 lgG2 *vs*. Week 12 HBsAg	0.10	-0.100, -0.289	0.34
Week 12 lgG3 *vs*. Week 12 HBsAg	0.13	-0.027, -0.295	0.10
Week 12 lgG4 *vs*. Week 12 HBsAg	0.05	-0.138, -0.229	0.62
Week24 lgG1 *vs*. Week24 HBsAg change from baseline	0.04	-0.161- 0.233	0.72
Week24 lgG2 *vs*. Week24 HBsAg change from baseline	0.05	-0.168 - 0.258	0.68
Week24 lgG3 *vs*. Week24 HBsAg change from baseline	0.03	-0.135- 0.199	0.71
Week24 lgG4 *vs*. Week24 HBsAg change from baseline	0.02	-0.177 - 0.223	0.82
Week24 lgG1 *vs*. Week24 HBsAg	0.01	-0.194 - 0.215	0.92
Week24 lgG2 *vs*. Week24 HBsAg	0.02	-0.199 - 0.238	0.86
Week24 lgG3 *vs*. Week24 HBsAg	0.01	-0.165 - 0.178	0.94
Week24 lgG4 *vs*. Week24 HBsAg	0.01	-0.205 - 0.210	0.98

### Value of Variables at 12 Weeks of PEG-IFN Treatment for Predicting HBsAg Clearance

Changes in week 12 IgG1, lgG2, lgG3, lgG4, HBsAg, and HBsAg levels from baseline were all strong predictors of HBsAg clearance. The largest AUC was the week 12 lgG3 (AUROC 0.867), followed by week 12 IgG1 (AUROC 0.852), week 12 lgG2 (AUROC 0.827), which were all better than week 12 HBsAg (AUROC 0.733) and HBsAg change form baseline (AUROC 0.711) (**Figure 4** and **Table 3**).

### Value of Variables at 24 Weeks of PEG-IFN Treatment for Predicting HBsAg Clearance

**Figure 5:**
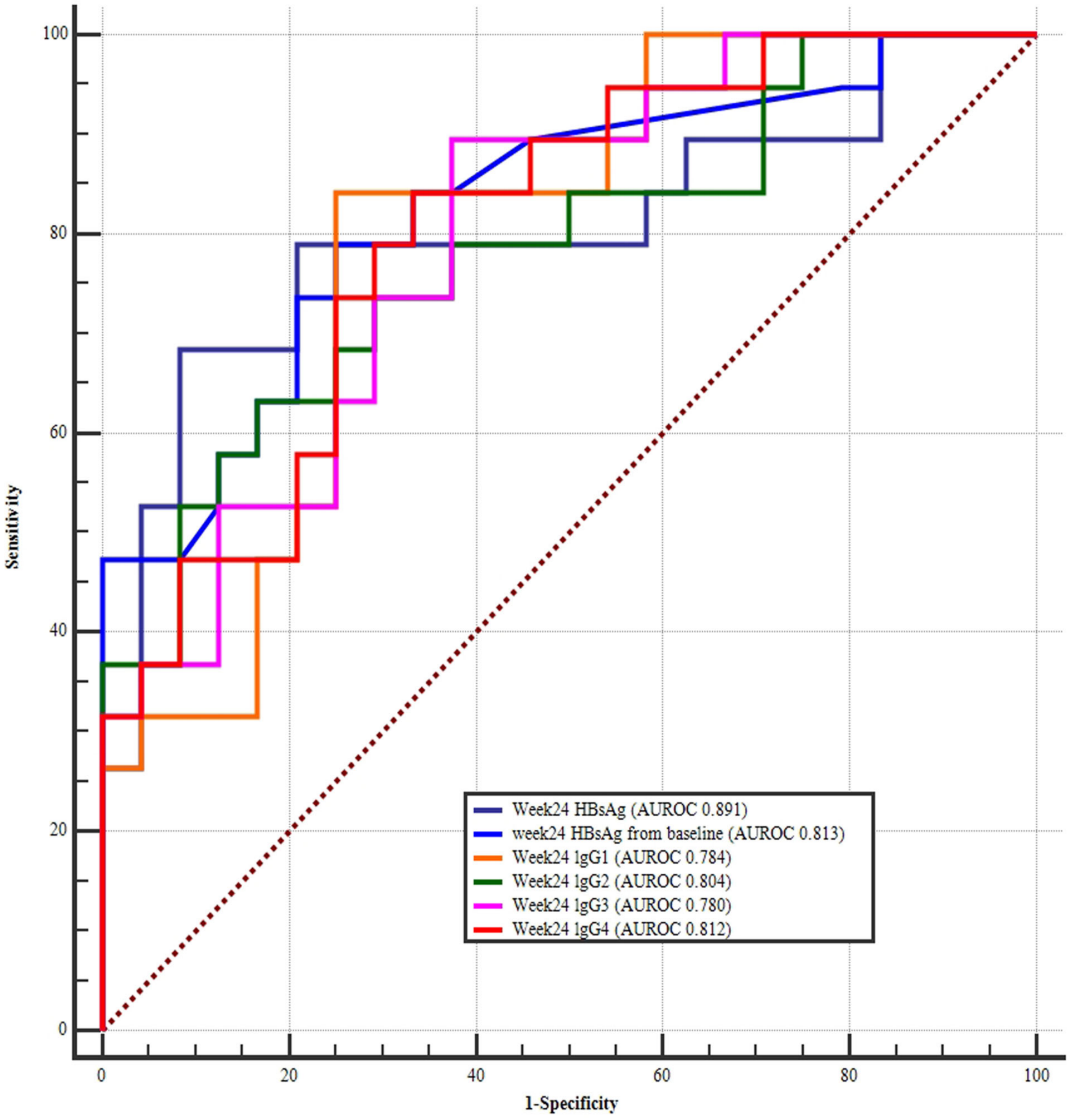
ROC curves of week 24 HBsAg, week 24 HBsAg change from baseline, week 24 IgG1, IgG2, IgG3, and IgG4 for predicting HBsAg clearance. The largest area under the curve was week 24 HBsAg (AUROC 0.891), followed by HBsAg change from baseline (AUROC 0.813), week 24 lgG4 (AUROC 0.812), week 24 lgG2 (AUROC 0.804), week24 lgG1 (AUROC 0.784), and week 24 lgG3 levels (AUROC 0.780).

The AUROC for the week 24 HBsAg levels (AUROC 0.891) was greatest in IHC patients treated with PEG-IFN interferon, followed by the HBsAg change from baseline (AUROC 0.813), and week 24 lgG4 (AUROC 0.812), week 24 lgG2 (AUROC 0.804), week 24 lgG1 (AUROC 0.784), and week 24 lgG3 (AUROC 0.780) levels (**Figure 5**). Further comparison of the AUROC indicated that even though the curves for HBsAg and HBsAg showed the changes from baseline were higher than the IgG1, IgG2, IgG3, and IgG4, but were not statistically different (**Table 3**).

### Multivariate Logistic Regression Analysis for Predicting HBsAg Clearance

**Figure 6:**
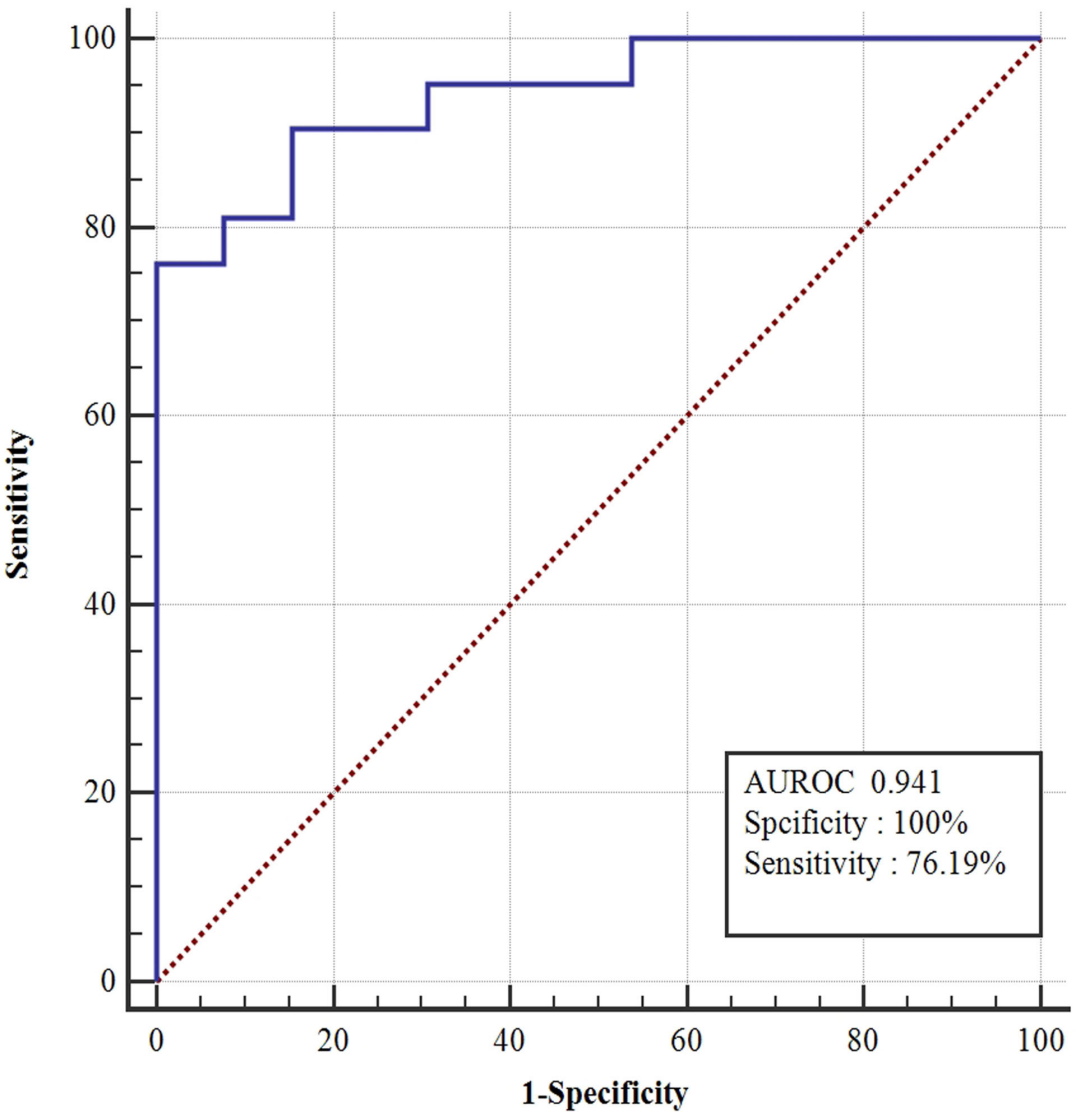
ROC curves of model (y=33.933-0.001*BaselinelgG1-0.002*BaselinelgG2). According to the univariate regression analysis, strong predictors included: baseline lgG1, lgG2, lgG3, and lgG4 levels; week 12 IgG1, lgG2, lgG3, and lgG4 levels; and week 24 IgG1, lgG2, lgG3, and lgG4 levels; HBsAg at 12 and 24 weeks; HBsAg change form baseline at 12 and 24 weeks. A multi-factor logistic analysis was performed with y=33.933-0.001*BaselinelgG1-0.002*BaselinelgG2. The area under the curve was up to 0.941 with 100% sensitivity and 76.19%.

According to the univariate regression analysis, strong predictors included: baseline lgG1, lgG2, lgG3, and lgG4 levels; week 12 IgG1, lgG2, lgG3, and lgG4 levels; and week 24 IgG1, lgG2, lgG3, and lgG4 levels; HBsAg at 12 and 24 weeks, HBsAg changes from baseline at 12 and 24 weeks. A multi-factor logistic analysis was performed with y=33.933-0.001*Baseline lgG1-0.002*Baseline lgG2. The AUROC was up to 0.941 with 100% sensitivity and 76.19% specificity. The optimal cutoff points of Baseline lgG1 was <15100ug/mL and Baseline lgG2 was <6750.67ug/mL respectively. (**Figure 6**).

## Discussion

The immunological mechanisms underlying HBV infection, particularly HBsAg clearance, are currently unclear. In this study, a cohort of IHCs treated with interferon was established, high HBsAg clearance rates were obtained, and blood specimens were dynamically retained, which provided the necessary basis for further work on mechanisms related to HBsAg clearance.

Persistent HBV infection can lead to impaired immune function in the body, and in addition to a decrease in the number and function of specific T cells, the humoral immune response is severely reduced. Previous studies have analyzed the relationship between the level of immunoglobulins in patients with chronic hepatitis B infection and the activity and severity of the disease and found that lgG levels are positively correlated with the severity of the disease (11, 12). After treatment with nucleoside analogs, HBV DNA levels decreased, and serum immunoglobulin level decreased accordingly (13, 14). However, it has not been reported how the immunoglobulin levels change after interferon treatment, particularly in HBsAg clearance patients. In this study, we investigated the changes of IgG and its subtypes in IHCs treated with interferon and analyzed their correlation with HBsAg clearance.

Several studies have confirmed that IHCs receiving IFN-based antiviral therapy can achieve higher HBsAg clearance rates. In this study, 60 IHCs were treated with IFN for 96 weeks, of which 39 patents achieved HBsAg clearance. During treatment, ALT and AST in the NR and R groups were increased, especially in HBsAg clearance group, which was similar to previous reports, suggesting that patients with good response to interferon therapy often experienced elevated ALT (15, 16). After treatment with PEG-IFN, the quantity of HBsAg at 12 weeks and 24 weeks was significantly lower than baseline, especially in the R group, suggesting that patients with rapidly declining HBsAg in the treatment had a higher incidence of HBsAg clearance (17). Therefore, many studies have indicated that changes in ALT and HBsAg at baseline and during treatment were good predictors of HBsAg clearance (15, 18, 19).

In this study, serum lgG1, lgG2, lgG3, and IgG4 levels were measured at baseline, and at 12 weeks, and 24 weeks of PEG-IFN treatment in IHCs. The levels of lgG1, lgG2, lgG3, and IgG4 were significantly lower in patients with HBsAg clearance than in non-cleared patients at all timepoints. Furthermore, we found the AUROC curves of serum IgG were significantly higher than HBsAg and ALT. This result suggests that serum IgG in the early stage of PEG-IFN treatment could be a good predictor of HBsAg clearance, and the decreased level of IgG is beneficial to HBsAg clearance. Multi-factor logistic regression analysis revealed that the combined baseline lgG1 and lgG2 levels had a significantly higher predictive value for HBsAg clearance with an AUROC of up to 0.941, a sensitivity of 100%, and a specificity of 76.19%.

This study provides evidence supporting serum IgG, as a clinical predictor of HBsAg clearance, which has a higher predictive value compared to the conventional predictors HBsAg and ALT levels. However, this study is a single-center exploratory study with a relatively small number of patients, and the exact predictive efficacy needs to be further validated in a larger sample size.

## Data Availability Statement

The raw data supporting the conclusions of this article will be made available by the authors, without undue reservation.

## Ethics Statement

The protocol and the consent form for the study were approved by the research ethical committee of the Beijing You’an Hospital, Capital Medical University, China ([2017]24). The patients/participants provided their written informed consent to participate in this study.

## Author Contributions

ZC designed research. HL and XL analyzed the results. LQ and YZ conducted the experiments. ZC and HL wrote the manuscript. XHL, XW, SL, YL, JZ, and XC reviewed the data. All authors contributed to the article and approved the submitted version.

## Funding

The authors disclose receipt of the following financial support for the research, authorship and/or publication of this article: Chinese National Natural Science Foundation (81900537), Beijing Hospitals Authority Clinical medicine Development of special funding support (XMLX202125), Capital Health Research and Development Projects (2020-1-2181), The Capital Characteristic Clinical Application Research (Z211100002921059).

## Conflict of Interest

The authors declare that the research was conducted in the absence of any commercial or financial relationships that could be construed as a potential conflict of interest.

## Publisher’s Note

All claims expressed in this article are solely those of the authors and do not necessarily represent those of their affiliated organizations, or those of the publisher, the editors and the reviewers. Any product that may be evaluated in this article, or claim that may be made by its manufacturer, is not guaranteed or endorsed by the publisher.

## Abbreviations

HBV, hepatitis B virus; HBsAg, hepatitis B surface antigen; IHCs, Inactive surface antigen carriers; ALT, alanine aminotransferase; HBeAg, hepatitis B e antigen; PEG-IFN, pegylated-interferon; lgG, immunoglobulin G; SD, standard deviation; ROC, Receiver operator characteristic; AUROC, area under the ROC curve.
