# Supplementary material for: Low Level of Serum Immunoglobulin G Is Beneficial to Clinical Cure Obtained With Pegylated Interferon Therapy in Inactive Surface Antigen Carriers
Source: Front Immunol. 2022 Apr 22;13:864354. doi: 10.3389/fimmu.2022.864354 (PMC9073012; doi:10.3389/fimmu.2022.864354)
Supplement: Supplementary file 1 [file Table_1.docx]

Supplemental table 1 Correlation between IgG and HBsAg in IHCs

|  | | lgG1 | lgG2 | lgG3 | lgG4 |
| --- | --- | --- | --- | --- | --- |
| Baseline HBsAg | r | .022 | .169 | .070 | .188 |
|  | p | .869 | .205 | .600 | .158 |
| 12week HBsAg | r | .190 | .106 | .296^*^ | .215 |
|  | p | .145 | .419 | .021 | .099 |
| 24week HBsAg | r | .251 | .380^**^ | .354^*^ | .177 |
|  | p | .097 | .010 | .017 | .244 |
